# Long non-coding RNA PVT1: A promising chemotherapy and radiotherapy sensitizer

**DOI:** 10.3389/fonc.2022.959208

**Published:** 2022-07-29

**Authors:** Weiping Yao, Shuang Li, Ruiqi Liu, Mingyun Jiang, Liang Gao, Yanwei Lu, Xiaodong Liang, Haibo Zhang

**Affiliations:** ^1^ Graduate Department, Bengbu Medical College, Bengbu, China; ^2^ Cancer Center, Department of Radiation Oncology, Zhejiang Provincial People’s Hospital, Affiliated People’s Hospital, Hangzhou Medical College, Hangzhou, China; ^3^ Graduate Department, Jinzhou Medical University, Jinzhou, China; ^4^ Cancer Center, Department of Medical Oncology, Zhejiang Provincial People’s Hospital, Affiliated People’s Hospital, Hangzhou Medical College, Hangzhou, China

**Keywords:** PVT1, lncRNA, chemoresistance, radioresistance, cancer

## Abstract

The long non-coding RNA (lncRNA) PVT1 was first found to activate variant translocations in the plasmacytoma of mice. Human lncPVT1 is located on chromosome 8q24.21, at the same locus as the well-known MYC oncogene. LncPVT1 has been found to promote the progression of various malignancies. Chemoresistance and radioresistance seriously affect tumor treatment efficacy and are associated with the dysregulation of physiological processes in cancer cells, including apoptosis, autophagy, stemness (for cancer stem cells, CSC), hypoxia, epithelial–mesenchymal transition (EMT), and DNA damage repair. Previous studies have also implicated lncPVT1 in the regulation of these physiological mechanisms. In recent years, lncPVT1 was found to modulate chemoresistance and radioresistance in some cancers. In this review, we discuss the mechanisms of lncPVT1-mediated regulation of cellular chemoresistance and radioresistance. Due to its high expression in malignant tumors and sensitization effect in chemotherapy and radiotherapy, lncPVT1 is expected to become an effective antitumor target and chemotherapy and radiotherapy sensitizer, which requires further study.

## Introduction

**Figure 2:**
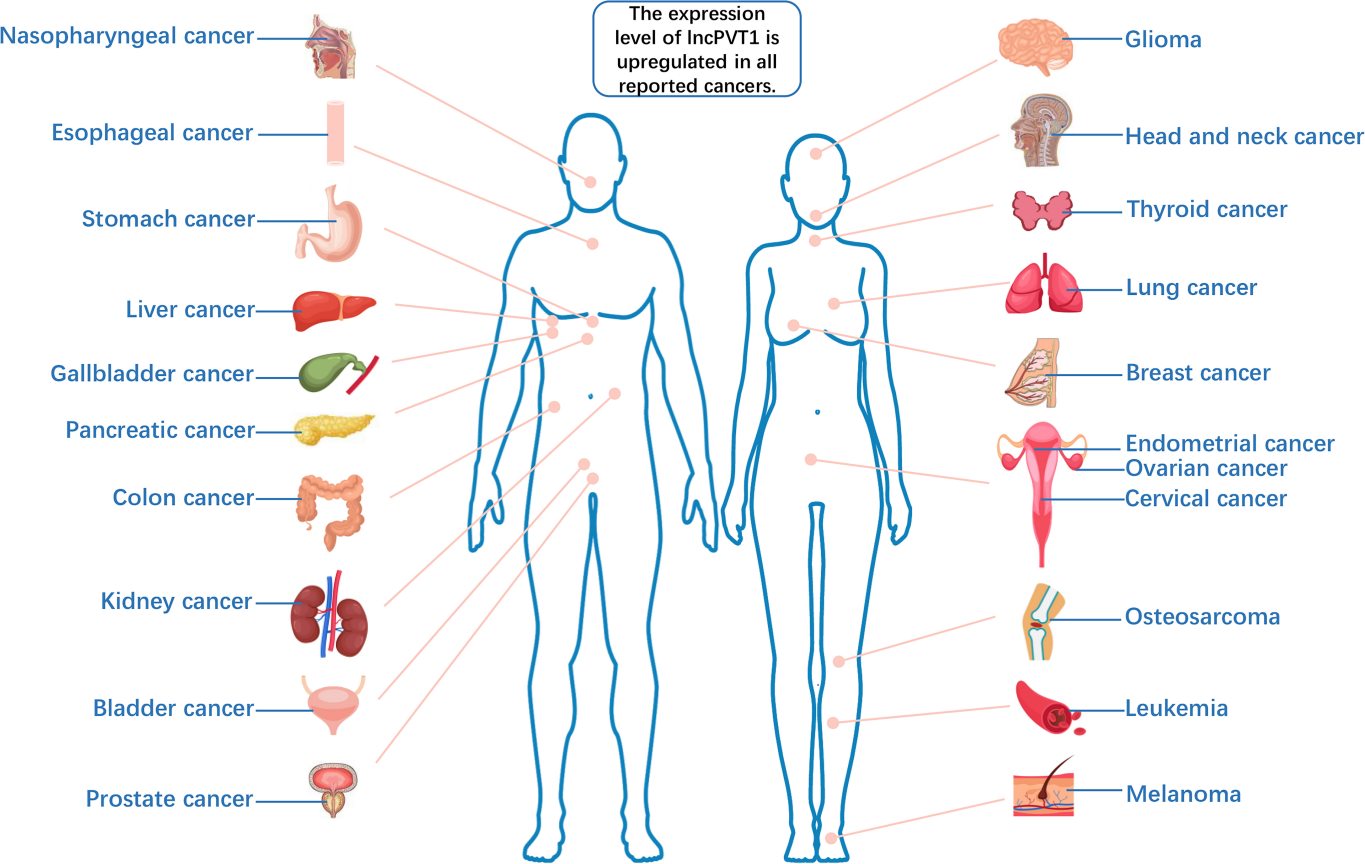
The expression level of lncPVT1 is upregulated in all reported cancers.

Long non-coding RNAs (lncRNAs), a class of functional RNA molecules larger than 200 nucleotides that cannot be translated into proteins, play an important regulatory role in epigenetics (1). LncPVT1 is an important member of the lncRNA family and was first found to activate variant translocations in the plasmacytoma of mice (2). Human lncPVT1 is located on chromosome 8q24.21, at the same locus as the well-known oncogene MYC (3) **(** **Figure 1** **)**. It not only interacts with MYC to promote cancer progression but also performs different oncogenic functions independent of MYC (4). LncPVT1 has been reported to be overexpressed in many types of malignant tumors, promoting cancer progression (5–9) (**Figure 2**). Moreover, lncPVT1 can regulate proliferation, invasion, autophagy, apoptosis, epithelial–mesenchymal transition (EMT), hypoxia, stemness (for cancer stem cells, CSC), exosomes, and other important physiological mechanisms (10–14).

**Figure:**
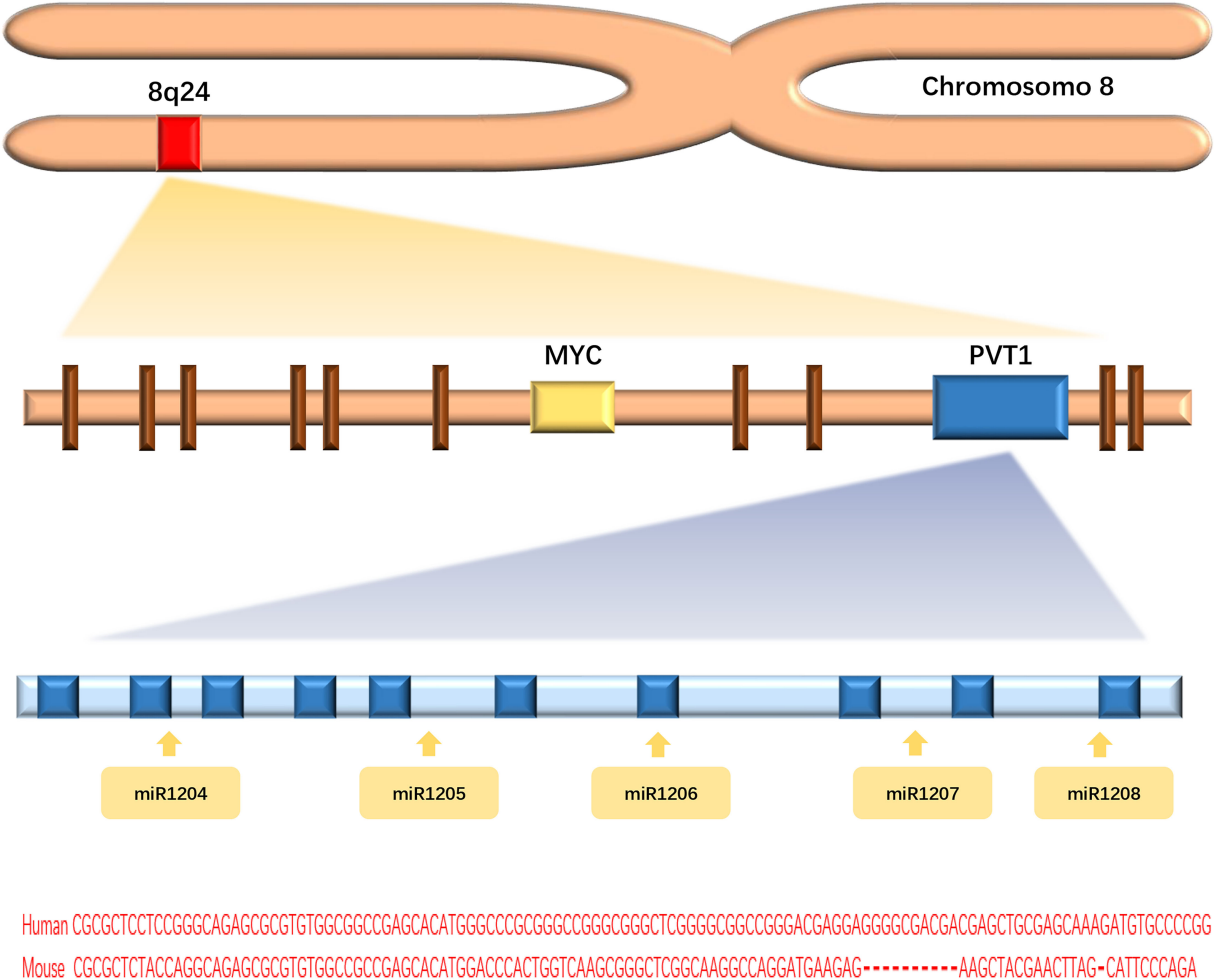
Figure 1 Graphical representation of the lncPVT1 genomic locus.

Chemotherapy is an important cancer treatment regimen that kills cancer cells primarily through the systemic or local use of chemosynthetic drugs and has a significant clinical benefit for patients (15). Chemoresistance leads to cancer recurrence and metastasis, hindering patient survival; hence, it remains the main obstacle in cancer treatment. Therefore, we need to understand the detailed regulatory mechanisms underlying chemoresistance to improve tumor cell sensitivity to chemotherapy (16). The currently reported molecular mechanisms associated with chemoresistance include the action of oncogenes, tumor suppressor genes, mitochondrial changes, DNA repair, autophagy, EMT, CSC, and exosomes (17). These pathways often intersect to increase the tolerance of tumors to cytotoxic drugs; therefore, the inhibition of these pathways will significantly improve the sensitivity of tumors to chemotherapy.

Radiotherapy is also an important form of cancer treatment. It can be used alone or in combination with other forms of treatment, either curative or palliative, for all stages of cancer. More than 50% of patients with cancer receive radiotherapy (18). Unfortunately, radioresistance reduces the efficacy of radiotherapy and seriously affects the quality of life of patients with cancer (19). Radiation therapy kills cancer cells directly or indirectly by causing DNA damage (20). However, ionizing radiation can also activate multiple prosurvival signaling pathways to promote DNA damage checkpoint activation, DNA repair, autophagy, apoptosis inhibition, and CSC. These signaling pathways conjointly protect cancer cells from radiation injury and promote radioresistance (21). Current literature indicates that the tumor microenvironment is closely related to radioresistance. Under hypoxic conditions, it has been shown that the radiosensitivity of tumor cells decreased significantly (22, 23).

LncPVT1 has gradually become a research hotspot regarding the regulation of chemoresistance and radioresistance, as studies have found that the regulatory mechanism of lncPVT1 in tumors is also associated with these processes. In recent years, lncPVT1 has been reported to regulate the chemoresistance of tumor cells through various pathways (**Table 1**). LncPVT1 silencing has also been shown to enhance radiosensitivity in nasopharyngeal carcinoma and lung cancer (**Table 2**). In this review, we summarize the various mechanisms through which lncPVT1 regulates chemosensitivity and radiosensitivity. We further emphasized that lncPVT1 is expected to be a new sensitizer of chemotherapy and radiotherapy and that it can be used to develop better clinical therapeutic strategies for patients with cancer.

**Table 1:** Molecular mechanisms underlying the lncPVT1-induced regulation of drug resistance.

Cancer	Pathway	Drug	References
Pancreatic cancer	miR-619-5p/Pygo2/Wnt/β-catenin axis; miR-619-5p/ATG14 axis	Gemcitabine resistance	(24)
	miR-409/SHH/GLI/MGMT axis	Gemcitabine resistance	(25)
	miR-143/HIF-1α/VMP1 axis	Gemcitabine resistance	(26)
	Curcumin regulates the PRC2/PVT1/C-MYC axis and then targets CSC	Gemcitabine resistance	(27)
	HAT1 binds EZH2 to prevent its degradation and promote lncPVT1 expression	Gemcitabine resistance	(28)
	Drosha and DGCR8 promote lncPVT1 to encode miR-1207-5p and miR-1207-3p, thereby regulating the expression of SRC and RhoA, respectively	Gemcitabine resistance	(29)
Gastric Cancer	mTOR/HIF-1α/P-gp and MRP1 pathway	Cisplatin (DPP) resistance	(30)
	Kanglaite (KLT) inhibits the expression of MDR1 and MRP1 by suppressing the expression of lncPVT1	Cisplatin (DPP) resistance	(31)
	miR-3619-5p/TBL1XR1 axis	Cisplatin (DPP) resistance	(32)
	Bcl2 activation to inhibit apoptosis	5-Fluorouracil resistance	(33)
Ovarian cancer	TGF-β1/p-Smad4/caspase-3 axis	Cisplatin (DPP) resistance	(34)
	miR-370/FOXM1 axis	Cisplatin (DPP) resistance	(35)
	JAK2/STAT3/PD-L1 axis	Cisplatin (DPP) resistance	(36)
Colorectal cancer	Inhibiting apoptosis and upregulating the expression of MDR1 and MRP1	Cisplatin (DPP) resistance	(37)
	Inhibiting apoptosis and upregulating the expression of MRP1, P-gp, mTOR, and Bcl-2	5-Fu resistance	(38)
Bladder cancer	Wnt/β-catenin axis	Doxorubicin (DOX) and Cisplatin (DDP) resistance	(39)
	MDM2/AURKB/p53 axis	Doxorubicin (ADM) resistance	(40)
Lung cancer	miR-216b/Beclin-1 axis	Cisplatin (DPP) resistance	(41)
	HIF-1α/miR-140-3p/ATG5/autophagy	Cisplatin (DPP) resistance	(42)
Breast Cancer	Preventing Nrf2 protein degradation by inhibiting the binding of Keap1 and Nrf2	Doxorubicin resistance	(43)
Osteosarcoma	miR152/c-MET/p-PI3K/p-AKT axis	Gemcitabine resistance	(44)
Glioma	Apoptosis pathways	Paclitaxel resistance	(45)
	miR-365/ELF4/SOX2 axis	Temozolomide (TMZ) resistance	(46)
Cervical cancer	Decreasing miR-195 expression by enhancing histone H3K27me3 in the miR-195 promoter region and also *via* direct sponging of miR-195	Paclitaxel (PTX) resistance	(47)
Head and neck squamous cell carcinoma (HNSCC)	By sponging miR-124-3p	Cetuximab resistance	(48)*
Prostate Cancer	Not mentioned	Castration resistance	(49)*

*LncPVT1 regulates resistance to immunotherapy and castration therapy.

**Table 2:** Molecular mechanisms underlying PVT1-induced radioresistance.

Cancer	Pathway	Mechanism	References
Nasopharyngeal carcinoma (NPC)	ATM/Chk2/p53 axis; caspase-9/caspase-7/PARP axis	DNA repair, apoptosis	(50)
	KAT2A/H3K9ac/TIF1β/NF90/HIF-1α axis	Hypoxic	(51)
	miR-515-5p/PIK3CA/p-AKT axis	Apoptosis	(52)
Non-small-cell lung cancer (NSCLC)	By sponging miR-195	Apoptosis	(53)
	miR-424-5p/CARM1 axis	Apoptosis	(54)

## PVT1 and Chemoresistance

### Pancreatic cancer

Pancreatic cancer is a highly malignant tumor with an extremely poor prognosis. The 1- and 5-year survival rates of this disease are only 24% and 9%, respectively (55). Gemcitabine is a first-line chemotherapeutic agent for advanced pancreatic cancer, and resistance to gemcitabine and other chemotherapeutic drugs is an important factor in the poor prognosis of this malignancy (56).

The expression level of lncPVT1 is significantly increased in pancreatic cancer. Moreover, lncPVT1 has been reported to promote the proliferation and migration of pancreatic cancer cells (13). Previous reports have suggested that decreased lncPVT1 levels can increase sensitivity of pancreatic cancer cells to gemcitabine chemotherapy (57, 58). Studies have shown that the activation of the Wnt/β-catenin pathway can induce chemoresistance in cancer cells (59). Autophagy is an important mechanism for tumor cell survival and has been proven to improve the tolerance of tumor cells to radiotherapy and chemotherapy (60). LncPVT1 competitively binds to microRNA-619-5p (miR-619-5p) to regulate the expression of Pygo2, mediating the Wnt/β-catenin pathway to increase the chemoresistance of pancreatic cancer cells to gemcitabine. At the same time, the autophagy-related protein ATG14 is also regulated by lncPVT1/miR-619-5p to increase autophagy, thereby promoting gemcitabine resistance in pancreatic cancer (24).

O6-Methylguanine-DNA methyltransferase (MGMT) is a DNA repair enzyme regulated by Wnt/β-catenin that protects cells from physicochemical damage by repairing damaged DNA (61). In addition, the Hedgehog (Hh) signaling pathway has been shown to be closely associated with tumor resistance and regulates autophagy (62, 63). Its terminal transcription factor Gli is responsible for transmitting signals into the nucleus and promoting transcriptional activation to upregulate the expression level of downstream target genes, MGMT (64, 65). Based on these studies, Yu Shi et al. found that lncPVT1 acts as a competing endogenous RNA (ceRNA) of miR-409 in pancreatic cancer cells. At the same time, miR-409 directly targets The Sonic Hedgehog (SHH) and regulates apoptosis and autophagy through the SHH/GLI/MGMT pathway, thus mediating the resistance of pancreatic cancer to gemcitabine (25). Furthermore, the hypoxia-inducible factor 1-alpha (HIF-1α)/vacuole membrane protein 1 (VMP1) axis mediates therapeutic resistance in colon cancer cells (66). Researchers recently found that lncPVT1 competitively binds to miR-143 and that decreased lncPVT1 levels and upregulated miR-143 levels increase the chemosensitivity of pancreatic cancer cells to gemcitabine through the HIF-1α/VMP1 axis (26).

In addition, lncPVT1 regulates the sensitivity of pancreatic cancer cells to gemcitabine by inhibiting enhancer of zeste homolog-2 (EZH2) at polycomb repressive complex 2 (PRC2), where EZH2 upregulates the expression of lncPVT1. LncPVT1, as an enhancer of MYC, promotes the expression of c-Myc, thus stimulating the activity of CSCs. The dysregulation of CSC in pancreatic cancer can induce chemoresistance (67). Another study has concluded that curcumin downregulates cancer stemness by inhibiting the PRC2/lncPVT1/c-MYC pathway, increasing the sensitivity of pancreatic cancer cells to gemcitabine (27). Sun et al. found that histone acetyltransferase 1 (HAT1) promotes the binding of bromodomain-containing 4 (BRD4) to the lncPVT1 promoter to enhance lncPVT1 expression. Simultaneously, HAT1 competitively binds to the N-terminus of EZH2 with the ubiquitin protein ligase E3 component n-recognin 4 (UBR4), preventing the ubiquitination of EZH2. Thus, HAT1 promotes gemcitabine resistance in pancreatic cancer cells (28). Based on this mechanism, tripolyphosphate (TPP)-siHAT1 nanoparticles have been developed to inhibit HAT1 expression and overcome gemcitabine resistance in pancreatic cancer cells. However, the efficacy and safety of TPP-siHAT1 nanoparticles require further validation.

You et al. have also discussed the mechanism of gemcitabine resistance in pancreatic cancer cells. They found that Drosha ribonuclease III (Drosha) and DGCR8 promote the lncPVT1-mediated processing of miR-1207-5p and miR-1207-3p. The generated products miR-1207-5p and miR-1207-3p inhibit sarcoma gene (SRC) and ras homolog family member A (RhoA), respectively, to increase the sensitivity of pancreatic cancer cells to gemcitabine chemotherapy (29). These studies highlight lncPVT1 as a promising target for improving the sensitivity of pancreatic cancer cells to gemcitabine.

### Gastric cancer

Gastric cancer remains the most common malignancy of the digestive system, ranking fifth in incidence and third in causing cancer-related deaths worldwide (68). Although radical resection is the standard treatment for early gastric cancer, patients are often diagnosed with gastric cancer at the advanced stage, making chemotherapy the main treatment regimen after diagnosis. Cisplatin, 5-FU, and other chemotherapeutic drugs are common first-line treatments for gastric cancer (69). However, the emergence of multi-drug resistance (MDR) reduces the sensitivity of chemotherapy and makes the survival benefit of patients with advanced gastric cancer worse (70).

Many studies have suggested that lncPVT1 can be used as a biomarker for the diagnosis and prognosis of gastric cancer (71). In addition, lncPVT1 can promote the growth and invasion of gastric cancer and induce angiogenesis (11, 72). The MDR-associated proteins include MDR1, mTOR, HIF-1α, and MRP. A decrease in lncPVT1 expression was found to increase the apoptosis rate of cisplatin-treated gastric cancer cell lines. Reverse transcription-quantitative polymerase chain reaction (RT-qPCR) and Western blotting (WB) showed that MDR1, mTOR, HIF-1α, and multi-drug resistance-associated protein 1 (MRP1) expression were all increased when lncPVT1 expression increased. The expression of these MDR-related genes promotes the expression of P-glycoprotein (P-gp). P-gp transports chemotherapeutic drugs out of the cell and helps the cell develop resistance to these drugs (73). Hence, lncPVT1 could be an effective target for reversing MDR in gastric cancer (30). Kanglaite (KLT) is a Chinese herbal formulation with antitumor effects (74), which is often combined with chemotherapy drugs to reduce the side effects of chemotherapy and increase chemosensitivity (75). Researchers found that KLT inhibited the expression of lncPVT1, reducing the expression of MDR1 and MRP1 in cisplatin-resistant gastric cancer cell lines (31).

Recently, the mechanism by which lncPVT1 regulates chemoresistance in gastric cancer has been further elaborated. Wu et al. found that lncPVT1 competes with miR-3619-5p to regulate the downstream target transducin beta-like 1 X-linked receptor 1 (TBL1XR1), regulating the sensitivity of gastric cancer cells to cisplatin (32). Moreover, 5-FU is also one of the main chemotherapeutic drugs for gastric cancer (76), so chemoresistance to 5-FU in gastric cancer deserves more attention. Du et al. found that lncPVT1 silencing could inhibit the expression of the anti-apoptotic protein Bcl-2, promoting apoptosis and inducing 5-FU resistance in gastric cancer cells (33). Therefore, lncPVT1 might play an important role in regulating cisplatin resistance in gastric cancer cells.

### Ovarian cancer

Ovarian cancer is the deadliest gynecological cancer and is a serious threat to women’s health worldwide. Approximately 70% of patients with this disease are already at an advanced stage upon diagnosis. Platinum-based chemotherapy and cytoreductive operations are standard treatments for ovarian cancer (77). Similarly, the clinical emergence of chemoresistance also poses great challenges for the survival of patients with this disease. Therefore, determining the target of chemoresistance in ovarian cancer is urgently needed.

LncPVT1 plays an oncogene role in ovarian cancer and promotes the proliferation and invasion of ovarian cancer cell (78, 79). Researchers found that lncPVT1 expression levels were significantly elevated in cisplatin-resistant ovarian cancer tissues (80). Liu et al. silenced lncPVT1 expression in cisplatin-resistant ovarian cancer cell lines and measured their cell viability and apoptosis rate after treatment with cisplatin. The results showed that the cell viability was significantly decreased and the apoptosis rate of tumor cells was significantly higher in downregulation of the lncPVT1 group than that of the control group after treatment with cisplatin. They also overexpressed lncPVT1 in cisplatin-sensitive ovarian cancer cell lines, which were then treated with cisplatin. Compared to the control group, cell viability in these lncPVT1-overexpressing cell lines was significantly increased, and the apoptosis rate was significantly reduced. RT-qPCR and WB results showed that the mRNA levels and protein expression of apoptosis-related genes TGF-β1, p-Smad4, and caspase-3 were significantly increased with a decrease in lncPVT1 expression level. Hence, lncPVT1 overexpression induces cisplatin resistance in ovarian cancer cells by inhibiting the apoptotic pathway (34).

Researchers further investigated how lncPVT1 regulates cisplatin resistance in ovarian cancer. Forkhead box M1 (FOXM1) has been reported to be involved in a variety of malignant behaviors of ovarian cancer cells, including growth, proliferation, invasion, and metastasis (72, 81). Yi et al. found that lncPVT1 acts as a molecular sponge for miR370 to inhibit miR370 and promote FOXM1 expression. Furthermore, lncPVT1 directly binds to FOXM1 and increases its protein expression level. Moreover, lncPVT1 promoted cisplatin resistance in ovarian cancer by increasing FOXM1 expression (35). Chen et al. found that lncPVT1 silencing can inhibit the growth and proliferation and promote apoptosis in cisplatin-resistant ovarian cancer cells by downregulating the JAK2/STAT3/PD-1 signaling pathway (36). The combination of lncPVT1-targeted therapy and PD-L1 immunotherapy is expected to improve the efficacy in patients with cisplatin-resistant ovarian cancer (82).

### Colorectal cancer

Colorectal cancer (CRC) is the third most common cancer worldwide. Cisplatin and 5-FU are the first-line chemotherapy agents for colorectal cancer (83). However, MDR is the main cause of treatment failure in patients with this disease. Researchers have found that lncPVT1 acts as a molecular sponge targeting miR-16-5p, thereby promoting proliferation, invasion, and migration of CRC cells by regulating VEGFA/VEGFR1/AKT signaling pathway (84). Moreover, lncPVT1/VEGFA axis promotes colon cancer metastasis and stemness by downregulation of miR-152-3p (10). LncPVT1 has also been found to promote proliferation, invasion, and migration of CRC cells by regulating the miR-106b-5p/FJX1 axis (85). Furthermore, lncPVT1 knockdown increased the apoptosis rate of cisplatin-resistant colorectal cancer cells, whereas the overexpression of lncPVT1 significantly enhanced the resistance of colorectal cancer cells to cisplatin. Moreover, silencing lncPVT1 increased the expression levels of pro-apoptotic proteins Bax and cleaved caspase-3 in cisplatin-resistant colorectal cancer cells while at the same time decreasing the expression levels of MDR1, MRP1, and the anti-apoptotic protein Bcl-2. We concluded that lncPVT1 regulates the sensitivity of colorectal cancer cells to cisplatin through the apoptotic pathway (37).

In addition, increased lncPVT1 expression promoted colorectal cancer cell tolerance to 5-FU. RT-qPCR and WB showed that lncPVT1 upregulated the expression levels of MRP1, mTOR, P-gp, and Bcl-2 (38). Therefore, lncPVT1 is an effective target for treating chemoresistance in colorectal cancer.

### Bladder cancer

Bladder cancer is a fatal malignancy of the urinary system that mainly affects men over 65 years of age. Surgical treatment, radiotherapy, chemotherapy, immunotherapy, and bladder perfusion therapy are used for patients with different stages of bladder cancer (86). As an oncogene of bladder cancer cells, lncPVT1 acts as a ceRNA targeting miR-194-5p to regulate the expression level of BCLAF1, thereby promoting the proliferation, migration, and anti-apoptosis of bladder cancer cells (87). A previous study showed that lncPVT1 promotes growth, migration, and invasion of bladder cancer by miR-31/CDK1 (88).

Researchers found a significant increase in lncPVT1 expression in bladder cancer cells resistant to doxorubicin (DOX) and cisplatin (DDP). Moreover, lncPVT1 silencing not only attenuated the growth, proliferation, and other malignant behaviors of drug-resistant bladder cancer cell lines but also increased their cell apoptosis rate and sensitivity to DOX and DDP. WB results showed that the expression levels of the drug-resistant related proteins MDR1 and MRP1 decreased with a decrease in lncPVT1 expression. Further studies on the regulatory mechanism of lncPVT1 revealed that lncPVT1 regulates the expression levels of drug-resistant related proteins MDR1 and MRP1 through positive regulation of the Wnt/β-catenin pathway, thus affecting the sensitivity of bladder cancer cells to DOX and DDP (39). In another study, Jiang et al. found that increased lncPVT1 expression promoted mouse double minute 2 (MDM2) and MDM2-mediated aurora kinase B (AURKB) expression. Subsequently, p53 ubiquitination is enhanced, increasing resistance to doxorubicin (ADM) in bladder cancer cells (40).

### Lung cancer

Lung cancer is the leading cause of cancer-related deaths worldwide, and smoking remains a major risk factor for this disease. Platinum-based chemotherapy is one of the main treatments for inoperable lung cancer (89); however, chemoresistance limits the application of platinum-based drugs in patients with lung cancer patients, increasing their mortality (90). Therefore, there is a need to study the mechanism of cisplatin tolerance in lung cancer and identify an effective target to solve this clinical problem.

LncPVT1 is highly expressed in non-small cell lung cancer (NSCLC) and is associated with poor prognosis (91). Wang et al. found that lncPVT1 facilitates the proliferation, migration, and invasion of NSCLC cells by indirectly mediating FGFR1 *via* targeting miR-551b (92). In another study, lncPVT1 activates the Wnt/β-catenin signaling pathway by miR-361-3p/SOX9 axis, thereby promoting the proliferation, migration, invasion, and anti-apoptosis of NSCLC cells (93). A previous study showed that lncPVT1 promotes angiogenesis by regulating the miR-29c/VEGF signaling axis in NSCLC (94). Beclin-1 is a biomarker for autophagy that has been reported to be involved in the malignant biological behavior of lung cancer cells (95). Chen et al. found that lncPVT1 sponges miR216b to inhibit Beclin-1 expression and induce cisplatin tolerance in NSCLC cells by regulating apoptosis and autophagy (41). Further studies have found that HIF-1α and other related pathways are activated to induce lncPVT1 expression under hypoxic conditions. The induction of lncPVT1 expression increases the expression level of ATG5 through competitive binding with miR140-3p and reduces the sensitivity of lung cancer cells to cisplatin by influencing the autophagy pathway (42). These studies indicate that lncPVT1 is expected to become a new target to solve the problem of chemoresistance in lung cancer and improve the efficacy of treatment for this disease.

### Breast Cancer

Breast cancer has replaced lung cancer as the most common malignant tumor worldwide. Triple-negative breast cancer (TNBC) is a subtype of breast cancer with negative estrogen receptor (ER), progesterone receptor (PR), and human epidermal growth factor receptor 2 (HER-2) expression. It is characterized by its invasiveness, aggressive metastasis, and recurrence (96). Chemotherapy is the standard treatment for TNBC; unfortunately, chemoresistance is a major obstacle in treating patients with breast cancer.

LncPVT1 promotes breast cancer cell proliferation, migration, invasion, and anti-apoptosis *via* regulating miR-543/TRPS1 axis (97). In TNBC cells cultured with mature adipogenic medium (MAM), lncPVT1 facilitates EMT, cell proliferation, and cell migration by regulating p21 expression (98). A recent study showed that lncPVT1 promotes cell migration and invasion by regulating miR-148a-3p/ROCK1 axis in breast cancer (99). The Keap1/Nrf2/ARE pathway is an important antioxidant stress signaling pathway in the body, which has been observed to regulate drug resistance in tumor cells (100, 101). Using bioinformatics analysis, Luo et al. concluded that lncPVT1 could interact with Keap1. They then conducted experiments to verify that lncPVT1 blocked the Keap1-mediated degradation of Nrf2 through competitive binding with Keap1. In turn, this binding increased the expression of Nrf2 and increased the expression of downstream drug-resistance-related molecules. Moreover, lncPVT1 increased adriamycin resistance in TNBC through this mechanism (43).

### Osteosarcoma

Osteosarcoma (OS) is a malignant tumor that usually occurs in children and adolescents. Patients with this disease often develop lung metastases. Surgical treatment, chemotherapy, and radiotherapy are the main therapeutic methods for OS (102, 103). In recent years, there have been breakthroughs in the application of immunotherapy in patients with OS (104). Chemotherapy treatments for OS often involve multi-drug combination therapy, usually including adriamycin, cisplatin, bleomycin, cyclophosphamide, and gemcitabine, among other drugs. Chemoresistance often leads to recurrence and metastasis in patients with osteosarcoma.

LncPVT1 plays a carcinogenic role in OS, promoting OS cell glucose metabolism, growth, proliferation, and invasion through regulating miR-497/HK2 axis (105). In a previous study, exosomes secreted by bone marrow mesenchymal stem cells (BMSCs) transfer lncPVT1 into osteosarcoma cells; the upregulation of lncPVT1 can promote ERG expression by inhibiting ERG ubiquitination and sponging miR-183-5p. LncPVT1 promotes the growth and metastasis of OS by regulating ERG expression (106). Moreover, lncPVT1 plays a potential role in regulating CSCs in OS (107). LncPVT1 is a ceRNA of miR-152. It activates the c-MET/PI3K/AKT pathway to enhance the resistance of OS cells to gemcitabine (44). Moreover, c-MET is a hepatocyte growth factor (HGF) receptor that has been reported to promote the progression of various cancers (108). The inhibition of c-MET has been shown to enhance the sensitivity of OS cells to cisplatin by inhibiting the PI3K/AKT pathway (109).

### Glioma

Glioma is the most common primary malignant brain tumor in adults. The treatment for this disease includes a combination of surgery, radiotherapy, and chemotherapy. Due to the blood–brain barrier and tumor heterogeneity, gliomas have strong drug resistance, leading to a high degree of malignancy and poor prognosis (110); hence, understanding the mechanisms of drug resistance in glioma is expected to improve patient prognosis.

LncPVT1 is highly expressed in gliomas, indicating poor prognosis. In addition, lncPVT1 can promote angiogenesis by regulating miR-1207-3p/hepatocyte nuclear factor 1β (HNF1B)/EMT axis in glioma (111). In another study, lncPVT1 influence bone morphogenetic protein (BMP) signaling pathway by regulating miR-128-3p/GREM1 axis, thereby promoting glioma cell proliferation, invasion, migration, and anti-apoptosis (112). A recent study showed that tumor suppressor gene p53 inhibits glioma cell proliferation, migration, and invasion while inducing apoptosis by blocking lncPVT1/TGF-β/Smad signaling pathway (113). Researchers knocked down lncPVT1 expression levels in SHG-44 glioma cells, added different concentrations of paclitaxel, and then measured their apoptosis rate. The results showed that the apoptosis rate of glioma cells was significantly higher in the lncPVT1-silenced group compared to the control group. These results suggest that decreased lncPVT1 expression increases the sensitivity of glioma cells to paclitaxel (45). Further research found that lncPVT1 acts as ceRNA to target miR-365 and positively regulate the expression level of E74-like ETS transcription factor 4 (ELF4). ELF4 has been reported to be highly expressed in glioma and can upregulate SOX2 expression to promote stemness (114). In conclusion, lncPVT1 facilitates stemness and TMZ resistance through regulating miR-365/ELF4/SOX2 signal axis in glioma (46).

### Cervical Cancer

Cervical cancer is the fourth most common cancer in women worldwide. Human papillomavirus (HPV) infection has been confirmed to be closely related to the occurrence and development of cervical cancer. HPV16, 18, 31, and 45 are the main causes of cervical cancer (115). Chemotherapy is the main treatment for advanced and recurrent cervical cancer, and platinum-based chemotherapy, such as cisplatin combined with paclitaxel, is often used. However, the emergence of chemoresistance significantly reduces the efficacy of this regimen in patients with cervical cancer.

It was observed that serum lncPVT1 levels in cervical cancer patients is generally elevated and correlated with cervical cancer tumor size, lymph node metastasis, and clinical stage. This suggests that lncPVT1 may be a novel serum biomarker for early diagnosis of cervical cancer (116). LncPVT1 acts as a ceRNA or sponge of miR-424 to promote the proliferation, migration, and invasion of cervical cancer cells (117). In addition, lncPVT1 has been found to regulate the sensitivity of cervical cancer cells to paclitaxel. Specifically, lncPVT1 directly and competitively binds to miR-195 and recruits EZH2 to induce histone H3 lysine 27 trimethylation (H3K27me3) in the miR-195 promoter region, thereby inhibiting the expression of miR-195. Downregulation of miR-195 promotes EMT in cervical cancer cells, thereby enhancing their chemoresistance to paclitaxel (47).

## PVT1 and Radioresistance

### Nasopharyngeal Cancer

Nasopharyngeal carcinoma (NPC) is a malignant tumor originating from the epithelial cells of the nasopharynx. This disease often occurs in southern China, with a significant regional concentration and ethnic susceptibility (118). Although the incidence of NPC is not high, NPC cells easily invade other tissues and metastasize, showing a strong degree of malignancy. NPC is closely related to Epstein–Barr virus infections (119). Radiotherapy has been established as the cornerstone of NPC treatment since 1965. However, in recent years, the development of intensity-modulated radiotherapy (IMRT) and proton radiotherapy (PRT) has brought significant survival benefits to patients with NPC (120). However, 10%–20% of NPC patients develop recurrence after radiotherapy due to radioresistance (121). Therefore, it is important to explore the molecular mechanism underlying radioresistance in NPC and search for new clues for radiotherapy sensitization in this disease (122).

In NPC, lncPVT1 has been found to promote cell proliferation and induce stemness by targeting miR-1207 (123). It was previously reported that the expression level of lncPVT1 was significantly upregulated in a radiation-induced mouse model, wherein RT-qPCR analysis showed that the expression level of lncPVT1 was significantly increased in the irradiated mice (124). Yi He et al. found that lncPVT1 silencing led to a decrease in phosphorylation of ATM/Chk2/p53 signaling pathway in NPC cells, which further led to the inhibition of the ATM-mediated homologous recombination (HR) repair pathway. Hence, more cells became apoptotic due to DNA damage that cannot be repaired in time (125). Meanwhile, decreased lncPVT1 expression levels after radiotherapy activated caspase, which is a core pro-apoptotic caspase, resulting in a cascade reaction that successively activates caspase-9, caspase-7, and PARP. PARP is believed to be the receptor for DNA damage and the cleavage substrate of caspase-7. Apoptosis is induced when activated caspase-7 binds to PARP (126). Moreover, the presence of lncPVT1 and MYC on the same chromosome band forms a positive feedback loop to promote tumor progression synergistically (3). In conclusion, lncPVT1 can promote DNA repair by phosphorylation of ATM/Chk2/p53 signaling pathway in NPC cells. LncPVT1 can also significantly inhibit apoptosis by blocking caspase-9/caspase-7/PARP axis, thereby leading to radioresistance (50).

Hypoxia induces radioresistance in tumor cells. HIF-1α is a major regulator of the hypoxia response and can be stably expressed under hypoxic conditions (127). Kyoto Encyclopedia of Genes and Genomes (KEGG) analysis showed that lncPVT1 was associated with the hypoxic phenotype. Because tumor hypoxia is common in NPC and it is associated with disease progression and resistance to therapy, Hong et al. suggested that targeting tumor hypoxia could be an effective approach for NPC treatment (128). Further studies revealed that lncPVT1 acts as a molecular scaffold for KAT2A and WDR5, coordinating their localization, and enabling KAT2A to acetylate H3K9 effectively. Acetylated H3K9 then recruits TIF1β to bind to chromatin, forming an H3K9ac/TIF1β complex that induces the transcription of NF90 and stabilizes HIF-1α expression (51). LncPVT1 induces radioresistance in NPC by activating the above-mentioned pathways and generating a hypoxic tumor microenvironment.

In addition, Han et al. found that lncPVT1 silencing inhibited cell proliferation, promoted cell apoptosis, and increased radiosensitivity in NPC cells. Moreover, lncPVT1 binds to miR-515-5p and negatively regulates its expression. An increase in lncPVT1 expression can reverse the inhibitory effect of miR-515-5p overexpression on the growth and proliferation of cancer cells and the promotive effect of miR-515-5p on the apoptosis and radiosensitivity of cancer cells. Meanwhile, miR-515-5p overexpression also reversed the inhibition of cyclin D1 expression and the elevation of Bax and cleaved caspase-3 levels. Further studies have shown that PIK3CA promotes growth and proliferation, inhibits apoptosis, and increases the radioresistance of NPC cells, whereas the overexpression of miR-515-5p reversed these effects. Moreover, miR-515-5p inhibition reversed the inhibitory effect of lncPVT1 silencing on the expression levels of PIK3CA and p-AKT. It has previously been reported that the p-AKT pathway is involved in regulating radioresistance in various cancers, and that PIK3CA can activate this pathway (129, 130). In conclusion, increased lncPVT1 expression activates the P-AKT pathway through the miR-515-5p/PIK3CA axis, thereby promoting cell proliferation, inhibiting apoptosis, and inducing radioresistance (52).

### Lung cancer

Patients with lung cancer typically receive a combination of treatments, but radiotherapy is the only treatment used for all types and stages of lung cancer. According to evidence-based models, 77% of patients with lung cancer are eligible for radiotherapy (131). With advancements in technology, radiotherapy has effectively improved treatment efficacy in patients with lung cancer while reducing the incidence of adverse reactions (132). However, radioresistance in lung cancer usually leads to recurrence and metastasis, which significantly reduces patient survival. Therefore, there is an urgent need to identify new targets for lung cancer radiotherapy sensitization to improve patient prognosis (133).

It has been reported that reduced lncPVT1 expression significantly inhibits the growth and proliferation of NSCLC cells. The apoptosis rate of NSCLC cells treated with a certain radiation dose was determined. The results showed that the apoptosis rate of the lncPVT1-silenced group was significantly higher than that of the control group, indicating that lncPVT1 silencing enhances the radiosensitivity of NSCLC cells. Luciferase reporter assays showed that lncPVT1 could directly interact with miR-195 and negatively regulate its expression. miR-195 inhibitors reversed the inhibitory effect of lncPVT1 silencing on cell proliferation and promoted apoptosis *in vitro*. In addition, miR-195 inhibitors also reversed the radiosensitization effect of lncPVT1 silencing in NSCLC cells. In conclusion, lncPVT1 acts as a molecular sponge of miR-195 in NSCLC, and the decreased expression level of lncPVT1 inhibits cell proliferation and promotes cell apoptosis, thus increasing the radiosensitivity of NSCLC cells (53).

Other related studies have shown that coactivator-associated arginine methyltransferase 1 (CARM1) can co-activate transcriptional regulation with PRMT1 and is overexpressed in NSCLC (134). CARM1 has been reported to be involved in regulating radiosensitivity in cervical cancer cells and colorectal cancer cells (135, 136). Wang et al. found that lncPVT1, as a ceRNA of miR-424-5p, promoted the expression of CARM1. The decrease in lncPVT1 levels or the increase in miR-424-5p levels inhibited the growth, proliferation, invasion, metastasis, and other malignant behaviors of NSCLC cells, promoted the apoptosis of NSCLC cells, and upregulated their radiosensitivity. RT-qPCR and WB showed that lncPVT1 silencing or miR-424-5p overexpression regulated the expression levels of MMP-2, MMP-9, Bcl-2, and Bax, which are related to tumor progression and apoptosis. Therefore, lncPVT1, as a ceRNA of miR-424-5p, regulates the radiosensitivity of NSCLC by regulating CARM1 (54).

## LncPVT1 and Resistance to other drugs

Head and neck squamous cell carcinoma (HNSCC) is the most common type of head and neck cancer and originates from the mucosal surface of the head and neck. Alcohol, tobacco, and HPV infection are risk factors for the induction of squamous cell carcinoma of the head and neck (137). EGFR is also usually highly expressed in HNSCC and is involved in regulating tumor occurrence and progression. Therefore, EGFR-targeted therapies are becoming increasingly popular for this disease. Cetuximab, a monoclonal antibody targeting EGFR, is often used in combination with radiotherapy to treat locally advanced HNSCC and has achieved good efficacy (138). LncPVT1 promotes laryngeal squamous cell carcinoma cell proliferation, migration, and anti-apoptosis by acting as a molecular sponge to regulate miR-519d-3p (139). In addition, lncPVT1 competitively combined with miR-150-5p to regulate GLUT1, thereby promoting the proliferation, migration, invasion, and anti-apoptosis of oral squamous cell carcinoma (140). LncPVT1 overexpression promoted HNSCC growth, significantly reduced the apoptosis rate of HNSCC cells treated with cetuximab, and reduced the drug sensitivity of HNSCC cells. As a tumor suppressor, miR-124-3p can reverse HNSCC tolerance to cetuximab. Furthermore, lncPVT1 can promote the resistance of HNSCC to cetuximab by inducing the methylation of the miR-124-3p promoter and downregulating miR-124-3p expression (48).

Prostate cancer is one of the most common malignant tumors in men worldwide, seriously affecting their health. Since androgens regulate the occurrence and progression of tumors, androgen-stripping therapy, specifically castration therapy, has become an important part of the prostate cancer treatment regimen. The clinical use of androgen inhibitors has significantly benefited patients with metastatic prostate cancer; however, the use of these drugs in metastatic castration-resistant prostate cancer (mCRPC), which occurs in some patients, remains a challenge (141). LncPVT1 could be used as a prognostic factor in patients with prostate cancer, representing poor survival (142). In prostate cancer, lncPVT1 has been found to promote EMT by regulating miR-186/Twist1 (143). LncPVT1 competitively binds to miR-15b-5p, miR-143-3p, miR-27a-3p, and miR-627-5p, then promotes prostate cancer invasion and metastasis by upregulating NOP2 (144). In another study, lncPVT1 promoted the viability of prostate cancer cells and inhibited their apoptosis by targeting miR-146a (145). Abiraterone is a second-generation androgen inhibitor used clinically for prostate cancer treatment. Abiraterone was used to treat lncPVT1-overexpressing prostate cancer cell lines and untreated pancreatic cancer cell lines *in vitro*. It was shown that lncPVT1 overexpression retained higher cell viability than the control group, suggesting that lncPVT1 induces castration resistance in prostate cancer cells (49).

## Conclusion

**Figure 3:**
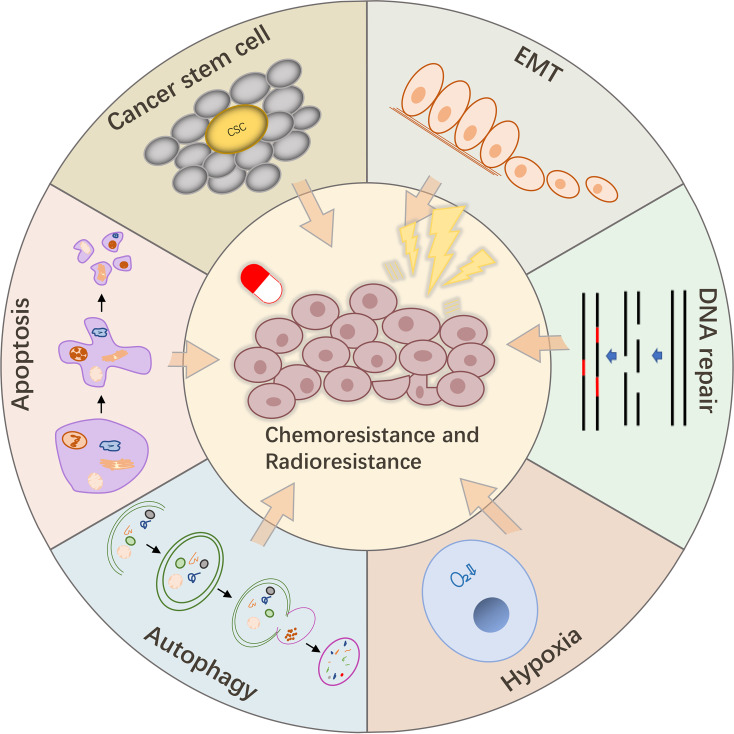
LncPVT1 regulates chemoresistance and radioresistance through autophagy, apoptosis, EMT, CSC, DNA damage repair, hypoxia, and other mechanisms.

Cancer treatment should be diversified to improve the survival of patients with various malignancies. As two powerful tools of cancer treatment, chemotherapy and radiotherapy have been shown to improve the survival of patients with cancer, which is of great significance (20, 146). However, the consequent chemoresistance and radioresistance have become the main obstacles to maintain the efficacies of these treatments. Hence, there is an urgent need to find new targets to overcome this barrier. The physiological mechanisms involved in the regulatory activities of lncPVT1 have many similarities with those involved in chemoresistance and radioresistance, including DNA damage repair, apoptosis, autophagy, EMT, stem cells, and hypoxia (**Figure 3**). This has led to extensive research on the relationship between lncPVT1, chemoresistance, and radioresistance (147, 148).

**Figure 4:**
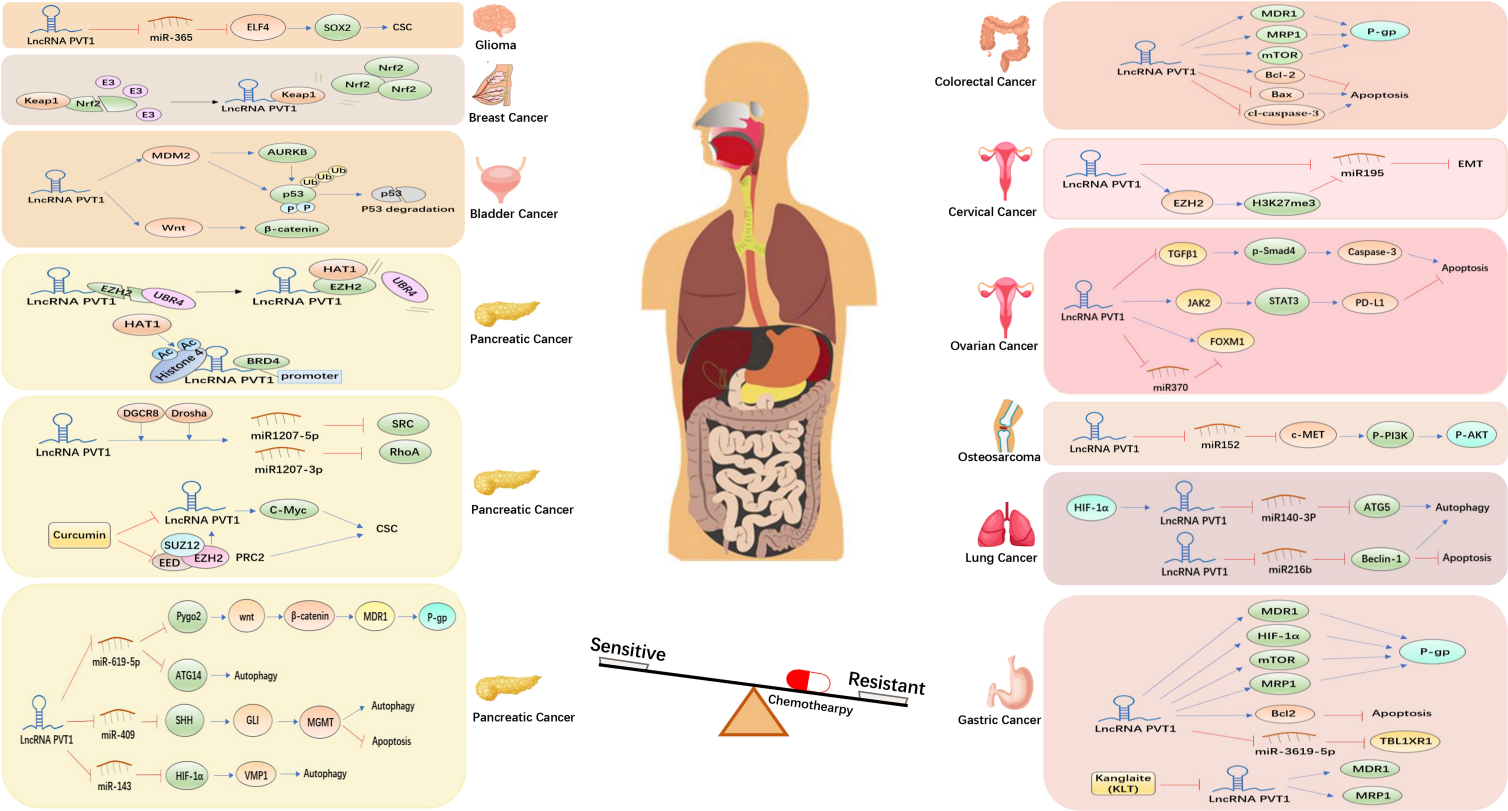
Molecular mechanisms underlying the lncPVT1-induced regulation of cancer chemoresistance.

**Figure 5:**
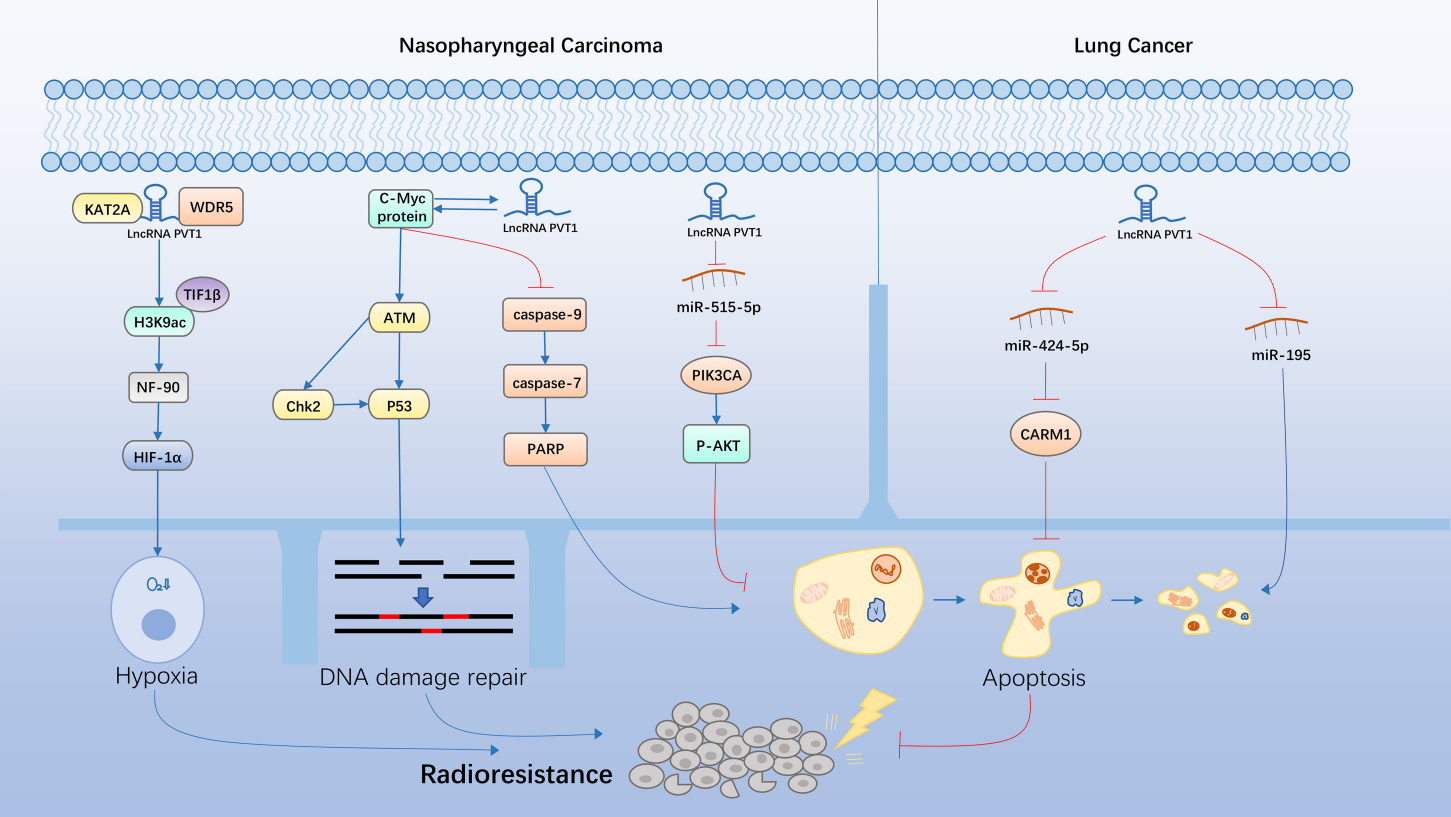
Molecular mechanisms underlying the lncPVT1-induced regulation of cancer radioresistance.

In recent years, several preclinical studies have explored the mechanisms underlying lncPVT1-induced chemoresistance and radioresistance in malignant tumor models (**Figures 4**, **5**). Among them, lncPVT1 can act as a molecular sponge of ceRNAs to inhibit miRNA expression, as a skeleton to bind proteins to specific targeted sequences, or bind proteins to form specific modules. In addition, lncPVT1 can encode miR-1204, miR-1205, miR-1206, miR-1207-5p, miR-1207-3p, and miR-1208 (149). Subsequently, downstream apoptosis and DNA damage repair pathways can be regulated to induce chemoresistance and radioresistance in cancer. Previous reviews have shown that lncPVT1 regulated various signaling pathways through miRNAs to influence cancer progression and chemoresistance (150). However, the types of miRNAs involved in the current study were limited. The interaction between lncPVT1 and other miRNAs needs to be revealed in a more comprehensive study of mechanisms related to chemoresistance and radioresistance. Many studies have only identified phenotypes in which lncPVT1 regulates chemoresistance or radioresistance in certain cancer cells, but a detailed analysis of the underlying regulatory mechanisms still needs to be performed. Hence, further research is required to address these gaps. Most therapeutic agents involved in the studies regarding lncPVT1 and chemoresistance are first-line treatments. Second- or third-line therapies are also commonly used in clinical oncology; therefore, it is necessary to investigate the effect of lncPVT1 expression on the efficacy of these therapeutic agents (151). So far, compared with chemoresistance, there have been few studies associating lncPVT1 and radioresistance, and the types of tumors involved are relatively few. Therefore, further research is needed on lncPVT1 and radiotherapy resistance.

Severe acute respiratory syndrome coronavirus 2 (SARS-CoV-2), as a new human coronavirus, has caused the coronavirus disease 2019 (COVID-19) pandemic, bringing significant harm and challenges to all parts of the world (152). In recent years, research on the link between COVID-19 and cancer has also increased. Patients with cancer, especially hematological malignancies, are at higher risk for breakthrough infections and severe consequences. The COVID-19 mRNA vaccine significantly reduces the risk of breakthrough infection in cancer patients (153). In addition, lncPVT1 has been reported to be involved in regulating SARS-CoV-2 infection (154). Therefore, the potential roles of lncPVT1 in cancer patients with COVID-19 is expected to be further investigated.

In conclusion, targeting lncPVT1 has broad therapeutic prospects in oncology. LncPVT1 inhibitors are expected to be developed in the future, which can be used in combination with radiotherapy and chemotherapy to reduce the incidence of chemoresistance and radioresistance and further improve the efficacy of these treatments in patients with malignancies. The detailed molecular mechanisms underlying the lncPVT1-induced regulation of chemoresistance and radioresistance require further study.

## Author contributions

All authors listed have made a substantial, direct, and intellectual contribution to the work, and approved it for publication.

## Funding

This study was supported in part by grants from the National Natural Science Foundation of China (82003236, to HZ), Zhejiang Provincial Nature Science Foundation of China (Grant number: LGF20H160030 to LG), and Zhejiang Health Science and Technology Project (Grant number: 2019KY280, to LG; 2022KY596, to HZ).

## Conflict of interest

The authors declare that the research was conducted in the absence of any commercial or financial relationships that could be construed as a potential conflict of interest.

## Publisher’s note

All claims expressed in this article are solely those of the authors and do not necessarily represent those of their affiliated organizations, or those of the publisher, the editors and the reviewers. Any product that may be evaluated in this article, or claim that may be made by its manufacturer, is not guaranteed or endorsed by the publisher.
